# Structural and functional studies of the glycoside hydrolase family 3 β-glucosidase Cel3A from the moderately thermophilic fungus *Rasamsonia emersonii*

**DOI:** 10.1107/S2059798316008482

**Published:** 2016-06-23

**Authors:** Mikael Gudmundsson, Henrik Hansson, Saeid Karkehabadi, Anna Larsson, Ingeborg Stals, Steve Kim, Sergio Sunux, Meredith Fujdala, Edmund Larenas, Thijs Kaper, Mats Sandgren

**Affiliations:** a Department of Chemistry and Biotechnology, Swedish University of Agricultural Sciences, Box 7015, 750 07 Uppsala, Sweden; b Department of Cell and Molecular Biology, Uppsala University, Box 596, 751 24 Uppsala, Sweden; c Laboratory for Protein Biochemistry and Biomolecular Engineering, Ghent University, Ledeganckstraat 35, B-9000 Ghent, Belgium; d DuPont Industrial Biosciences, 925 Page Mill Road, Palo Alto, CA 94304, USA

**Keywords:** glycoside hydrolase, β-glucosidase, biodegradation, crystal structure, *Rasamsonia emersonii*, Cel3A, glycoproteins, thermophilic fungus

## Abstract

The filamentous fungus *Hypocrea jecorina* produces a number of cellulases and hemicellulases that act in a concerted fashion on biomass and degrade it into monomeric or oligomeric sugars. β-Glucosidases are involved in the last step of the degradation of cellulosic biomass and hydrolyse the β-glycosidic linkage between two adjacent molecules in dimers and oligomers of glucose. In this study, it is shown that substituting the β-glucosidase from *H. jecorina* (*Hj*Cel3A) with the β-glucosidase Cel3A from the thermophilic fungus *Rasamsonia emersonii* (*Re*Cel3A) in enzyme mixtures results in increased efficiency in the saccharification of lignocellulosic materials. Biochemical characterization of *Re*Cel3A, heterologously produced in *H. jecorina*, reveals a preference for disaccharide substrates over longer gluco-oligosaccharides. Crystallographic studies of *Re*Cel3A revealed a highly N-glycosylated three-domain dimeric protein, as has been observed previously for glycoside hydrolase family 3 β-glucosidases. The increased thermal stability and saccharification yield and the superior biochemical characteristics of *Re*Cel3A compared with *Hj*Cel3A and mixtures containing *Hj*Cel3A make *Re*Cel3A an excellent candidate for addition to enzyme mixtures designed to operate at higher temperatures.

## Abstract 2

Cel3A from the thermophilic fungus *R. emersonii* has proven to be more efficient in the hydrolysis of β-glycosidic linkages than Cel3A from *H. jecorina*.

## 1. Introduction   

The complete degradation and saccharification of cellulose requires a suite of synergistically acting enzymes. Retaining β-glucosidases (BGLs) belong to glycoside hydrolase (GH) families GH1, GH3, GH5, GH30 and GH116 (Lombard *et al.*, 2014 ▸). They hydrolyse the β-linkage from the reducing end of glucose oligosaccharides. These enzymes are secreted by cellulose-degrading organisms and it has been shown that enzyme mixtures with enhanced levels of the native GH3 Cel3A from the mesophilic fungus *Hypocrea jecorina* (*Hj*Cel3A) benefit the conversion of cellulose to glucose. The GH3 Cel3A from the thermophilic fungus *Rasamsonia emersonii* (*Re*Cel3A) is a more efficient additive to enzyme mixtures compared with *Hj*Cel3A. Biochemical characterization of *Re*Cel3A revealed a substrate preference for disaccharides over longer oligosaccharides. The crystal structure of *Re*Cel3A is a tetramer composed of two biological dimers. Each protein molecule has a three-domain architecture, as observed for previous glycoside hydrolase family 3 BGLs. Interesting features of the structure are the long C-terminal linker that extends the active-site cleft and the high degree of N-glycosylation. There are N-glycan chains that are partially covered by the extended linker and N-glycans that comprise part of the dimeric interface.

### 1.1. The need for fuels from biomass   

The production of biofuels and chemicals in biorefineries from biomass, in lieu of nonrenewable petrochemicals, has garnered much attention in recent years (Ragauskas *et al.*, 2006 ▸; Kamm & Kamm, 2007 ▸). Cellulose is a major structural polysaccharide in plant cell walls and is a highly attractive renewable energy source as it is the most abundant polysaccharide on earth (Chundawat *et al.*, 2011 ▸). Cellulose is built up of β-1,4-linked glucose molecules and can be degraded to the monosaccharide glucose using enzymes. In the cell wall, cellulose molecules are organized into fibrils in which the chains are parallel to each other. Intermolecular hydrophobic and hydrophilic interactions hold the cellulose chains in a fibril together. Cellulose fibrils are highly recalcitrant and are not readily accessible to microbial and enzymatic degradation (Himmel *et al.*, 2007 ▸). The filamentous fungus *H. jecorina* is capable of producing large amounts of extracellular plant polysaccharide-degrading enzymes (Martinez *et al.*, 2008 ▸) and these enzymes have been used for a wide variety of industrial applications (Nakari-Setälä *et al.*, 2009 ▸). To degrade lignocellulosic biomass *H. jecorina* produces a set of cellulases, which work together synergistically to degrade the recalcitrant cellulose polymer. The three main groups of cellulose-degrading enzymes are endoglucanases [endo-(1,4)-β-d-glucanhydrolases; EC 3.2.1.4], which randomly cleave the β-1,4 linkage between two adjacent glucose units in the cellulose polymer, cellobiohydrolases [(1,4)-β-d-glucan cellobiohydrolases; EC 3.2.1.91], which processively release the disaccharide cellobiose from either the reducing or the non­reducing end of a polymer, and BGLs (EC 3.2.1.21), which hydrolyse cellobiose into glucose monosaccharides.

### 1.2. Improvement of cellulase mixtures: ratio optimization, protein engineering and enzyme-homology screen   

The enzymes needed for the industrial production of biofuels from lignocellulosic biomass represent a significant part of the process costs. Therefore, there is interest in obtaining enzyme mixtures with increased performance. One approach is to optimize the enzyme ratios of the mixture. It has been shown that enriching the *H. jecorina* secretome with additional amounts of the endogenous BGL Cel3A (*Hj*Cel3A) increases the performance of the mixture in the conversion of cellulose to glucose (Karkehabadi *et al.*, 2014 ▸; Barnett *et al.*, 1991 ▸). Alternatively, enzyme cocktails can be improved by protein engineering (Lantz *et al.*, 2010 ▸). An approach for further improving the enzyme mixtures is to substitute components with homologues from alternative sources.

The thermophilic fungus *R. emersonii* (formerly called *Talaromyces emersonii*) lives in soil and compost heaps and produces a complete set of cellulose-degrading enzymes (Folan & Coughlan, 1978 ▸). A number of *R. emersonii* cellulose-degrading enzymes have previously been characterized (Coughlan *et al.*, 1984 ▸; Moloney *et al.*, 1983 ▸; McHale, 1987 ▸), including four endoglucanases and three cellobiohydrolases. In addition, four *R. emersonii* BGLs have been identified and expressed (McHale & Coughlan, 1981 ▸, 1982 ▸; Coughlan & McHale, 1988 ▸), of which three are GH3 enzymes: a β-xylosidase (Bxl1), an avenacinase (Aven1; Morrison *et al.*, 1990 ▸; Reen *et al.*, 2003 ▸; Murray *et al.*, 2004 ▸; Collins *et al.*, 2007 ▸) and a BGL (Cel3A), which is the subject of this study. The latter has been biochemically characterized in a study by Murray *et al.* (2004 ▸).

Within the classification system of carbohydrate-active enzymes, CAZY (Lombard *et al.*, 2014 ▸), retaining BGLs can be found in glycoside hydrolase (GH) families GH1, GH3, GH5, GH9, GH116 and GH30. Except for those in GH9, these BGLs perform hydrolysis using a double-displacement reaction mechanism with net retention of configuration at the anomeric C atom (Gebler *et al.*, 1992 ▸). The structure of all BGLs is an (α/β)_8_ TIM barrel, but in GH9 they exhibit an (α/α)_6_ fold. Beside BGLs, the very large GH3 family, currently containing over 6300 proteins, also includes enzymes with the following catalytic activities: β-d-xylopyranosidases, *N*-acetyl-β-d-glucosaminidases (NagZs), α-l-arabinofuranosidases and exo-1,3- and exo-1,4-β-glucanases, as well as several activities involving the hydrolysis of glycoconjugates containing at least a single pyranose molecule.. The first GH3 crystal structure, presented in 1999, was the structure of barley (*Hordeum vulgare*) β-d-glucan exohydrolase (*Hv*ExoI; Varghese *et al.*, 1999 ▸). The complete *Hv*ExoI structure exhibits two domains: an (α/β)_8_-barrel (TIM barrel) and an (α/β)_6_ sheet (β-sandwich). This two-domain structure is common to all GH3s except for a subset of bacterial GH3s which exhibit a single (α/β)_8_-barrel domain (NagZs; Litzinger *et al.*, 2010 ▸; Bacik *et al.*, 2012 ▸). After *Hv*ExoI, an additional seven GH3 BGL structures have been reported to date: Bgl3B from the thermophilic bacterium *Thermotoga neapolitana* (*Tn*Bgl3B; Pozzo *et al.*, 2010 ▸), BglI from the yeast *Kluyveromyces marxianus* (Yoshida *et al.*, 2010 ▸), ExoP from the marine bacterium *Pseudoalteromonas* sp. BB1 (Nakatani *et al.*, 2012 ▸), BGL1 from the filamentous fungus *Aspergillus aculeatus* (*Aa*BGL1; Suzuki *et al.*, 2013 ▸), two β-glucosidases from *A. fumigatus* (*Af*βG) and *A. oryzae* (*Ao*βG) (Agirre *et al.*, 2016 ▸) and Cel3A/Bgl1 from the filamentous fungus *H. jecorina* (Karkehabadi *et al.*, 2014 ▸).

In this study, *Re*Cel3A was cloned and expressed heterologously in *H. jecorina* and subsequently purified for crystallization and biochemical characterization. *Re*Cel3A is an efficient cellobiase. We compared the efficiency of the hydrolysis of lignocellulosic biomass by mixtures that contained either *Re*Cel3A or Cel3A from *H. jecorina* (*Hj*Cel3A). Detailed biochemical analysis revealed that mixtures containing *Re*Cel3A yielded a significantly improved performance. We also present the three-dimensional crystal structure of Cel3A from the moderately thermophilic filamentous fungus *R. emersonii* solved to 2.2 Å resolution. The structure was solved with an intact extensive C-terminal loop, similar to those observed for *Af*βG and *Ao*βG (Agirre *et al.*, 2016 ▸) but different compared with the partially flexible or proteolytically cleaved C-terminal loop observed in the structure of *Aa*BGL1 (Suzuki *et al.*, 2013 ▸). In addition, it exhibits extensive N-glycosylation.

## 2. Methods   

### 2.1. Expression and purification of *R. emersonii* Cel3A   

The *cel3a* gene from *R. emersonii* (GenBank AAL69548.3) was codon-optimized for expression in *H. jecorina* and synthesized by GeneArt (now LifeTechnologies, Grand Island, New York, USA). The synthetic gene was cloned into a pTrex3G shuttle vector (amdS^R^, amp^R^, P_cbh1_; Foreman *et al.*, 2005 ▸). This construct was then used for the transformation of a derivative of *H. jecorina* strain RL-P37 with the four major cellulases deleted (*cel5A*, *cel6A*, *cel7A* and *cel7B*; Foreman *et al.*, 2005 ▸). Transformants of *H. jecorina* were picked from Vogel’s minimal medium plates (Vogel, 1956 ▸) containing acetamide after 7 d incubation at 37°C. Picked transformants were grown in Vogel’s minimal medium with a mixture of glucose and sophorose as a carbon source. The resulting *H. jecorina* strain expressed *Re*Cel3A at levels of greater than several grams per litre, constituting more than 50% of the total secreted protein, as judged by SDS–PAGE. The supernatant was concentrated to 168 g of total protein per litre by ultrafiltration at 4°C using Vivaspin 20 centrifuge concentration tubes with 3000 Da molecular-mass cutoff (Sartorius Stedim Biotech, France).

The *Re*Cel3A culture liquid was sterile-filtered (Sarstedt Filtropur 0.2 µm filters) and then purified on an ÄKTA­explorer (GE Healthcare Biosciences, Sweden) by gel filtration using a Superdex 200 16/60 GL column (GE Healthcare Biosciences, Sweden). The column was equilibrated with 25 m*M* bis-tris propane pH 7.5. Elution fractions containing *Re*Cel3A were concentrated using Vivaspin 20 centrifuge concentration tubes with 3000 Da molecular-mass cutoff (Sartorius Stedim Biotech, France) to a concentration of 15 mg ml^−1^ for enzyme-crystallization studies. The *Re*Cel3A protein was further purified by affinity chromatography using a *p*-aminobenzyl-thio-β-glucopyranoside tag coupled to activated Sepharose (GE Healthcare, Uppsala, Sweden) according to the manufacturer’s instructions. The affinity column was equilibrated and washed with 100 m*M* acetate buffer pH 5.0 containing 200 m*M* NaCl. The bound protein was eluted from the column with 100 m*M* glucose in 100 m*M* acetate buffer pH 5.0. Glucose was removed by repeated concentration and dilution using the Vivaspin 20 tubes mentioned above. The *Re*Cel3A sample was highly pure as judged by SDS–PAGE after the affinity-chromatography purification. The purified samples were used for kinetic analyses. The protein concentration was estimated by measuring the absorbance of the protein solution at 280 nm using a calculated extinction coefficient of 165 630 *M* ^−1^ cm^−1^ for *Re*Cel3A.

### 2.2. Enzyme kinetics of *Re*Cel3A   

Kinetic characterization of *Re*Cel3A was carried out using the substrates 2-chloro-4-nitrophenyl-β-d-glucopyranoside (CNPG) and 4-nitrophenyl-β-d-glucopyranoside (pNPG) (Sigma–Aldrich, USA). Both assays were run at 37°C in 100 m*M* phosphate buffer pH 5.0 in Eppendorf tubes incubated in a Thermomixer R (Eppendorf, Germany). Enzyme at a suitable concentration (1–0.5 n*M*) was added for single measurements in each experiment to 600 µl substrate solution. At each time point, 100 µl of reaction mixture was withdrawn and added to 100 µl 0.5 *M* Na_2_CO_3_. The absorbance of the sample was then measured at 415 nm in a spectrophotometer. The initial velocity {[CNP] (µ*M* min^−1^) and [pNP] (µ*M* min^−1^)} was calculated using a standard curve for CNP and pNP in the range 0–30 µ*M*. The kinetic parameters were calculated by fitting the data to the Michaelis–Menten equation with *Plot* (Wesemann, 2007 ▸). Using the two natural substrates cellobiose and cellotriose, the reaction was followed by detecting the catalytic products using high-performance anion-exchange chromatography with pulsed amperometric detection (HPAEC-PAD; Dionex ICS-3000, Sunnyvale, California, USA). A solution with a substrate concentration of 50–3000 µ*M* and enzyme at 1.4–0.6 n*M* concentration was incubated at 37°C at pH 5.0. An aliquot of 30 µl of the sample was withdrawn and added to 30 µl of 0.1 *M* NaOH to stop the reaction and this was performed at 2 min intervals for 10 min. Each sample was then loaded onto a CarboPac PA-100 analytical column (4 × 250 mm; Dionex, Sunnyvale, California, USA). Elution was performed using 100 m*M* NaOH and a gradient of sodium acetate from 10 to 170 m*M* in 100 m*M* NaOH over 27 min at a flow rate of 1 ml min^−1^. Quantification of the hydrolysis products was performed using standards for the hydrolytic products.

### 2.3. Differential scanning calorimetry   

*Re*Cel3A samples were dialyzed against 10 m*M* sodium acetate pH 5.0. The samples were diluted to 0.5 mg ml^−1^ in the absence and presence of 1 m*M* glucose. The heat capacity was recorded over a temperature trajectory of 30–100°C at a scan rate of 200°C h^−1^ using a MicroCal VP-Capillary DSC microcalorimeter (GE Healthcare, Pittsburgh, Pennsylvania, USA). Unfolding was irreversible for all tested samples.

### 2.4. Saccharification assay   

Corn stover was pretreated with dilute sulfuric acid by the US Department of Energy National Renewable Energy Laboratory (NREL). It was washed with water and the pH was adjusted to 5.0 using soda ash. The acid-pretreated corn stover contained 56% cellulose, 4% hemicellulose and 29% lignin. Enzymes were dosed based on total protein load, and total protein was measured using either a bicinchoninic acid (BCA) assay kit (Bio-Rad, Hercules, California, USA) or the biuret method (Lowry *et al.*, 1951 ▸). The enzyme was dosed as milligrams of protein per gram of cellulose in the reaction. Various amounts of *Re*Cel3A (0.1–10 mg g^−1^) in an experimental setup with four replicates were added to a base level of 10 mg g^−1^ P37 Δ*bgl1*, which is *H. jecorina* strain P37 with the *bgl1* (*cel3A*) gene deleted. 75 µl of pretreated corn stover (PCS, loading 7% cellulose) per well was loaded into a flat-bottom 96-well microtitre plate (MTP). 30 µl of appropriately diluted enzyme solution was added to each reaction well. The plates were covered with aluminium plate sealers and incubated in a plate incubator at 50°C with shaking. The reaction was terminated after 48 h incubation by adding 100 µl 100 m*M* glycine pH 10. After thorough mixing, the reaction mixtures were filtered through a 96-well filter plate (0.45 mm, PES; Millipore, Billerica, Massachusetts, USA). The filtrate was diluted into a plate containing 100 µl 10 m*M* glycine pH 10.0, and the amount of soluble sugars produced was measured by HPLC (Agilent 1100, Agilent, Santa Clara, California, USA) equipped with a de-ashing guard column (catalogue No. 125-0118, Bio-Rad, Hercules, California, USA) and a lead-based carbohydrate column (Aminex HPX-87P, Medway, Massachusetts, USA). The mobile phase was water and the flow rate was 0.6 ml min^−1^. The fractional cellulose conversion was calculated from the amounts of released glucose and cellobiose divided by the maximum possible amount of glucose that can be produced. The amounts of cellobiose were corrected for the weight of one extra water molecule upon hydrolysis to glucose.

### 2.5. Crystallization and data collection   

Crystallization of *Re*Cel3A was carried out at 293 K using the hanging-drop vapour-diffusion method (McPherson, 1999 ▸). Crystallization drops were produced by mixing 1 µl 18.9 mg ml^−1^ protein solution in 25 m*M* bis-tris propane (Sigma–Aldrich, USA) pH 7.5 with 1 µl reservoir solution consisting of 0.15 *M* MgCl.6H_2_O (Merck Millipore, Germany), 16%(*w*/*v*) polyethylene glycol (PEG) 3350 (Hampton Research, USA). Rectangular crystals appeared in the drops within one week. Prior to X-ray data collection, crystals of *Re*Cel3A were transferred to a cryoprotectant solution consisting of 40%(*v*/*v*) 2-methyl-2,4-pentanediol (MPD; Hampton Research, USA) and 60%(*v*/*v*) reservoir solution before flash-cooling them in liquid nitrogen. X-ray diffraction data for *ReCel3A* were collected on the ID23-1 beamline at the European Synchrotron Radiation Facility (ESRF), Grenoble, France.

### 2.6. Structure determination and refinement   

The collected X-ray data were processed using *XDS* (v. February 3, 2010; Kabsch, 2010 ▸) and scaled using *SCALA* (v.3.3.16; Winn *et al.*, 2011 ▸). Initial phases were obtained using the molecular-replacement (MR) method in *Phaser* (v.2.1.4; McCoy *et al.*, 2007 ▸) using *Hj*Cel3A (PDB entry 3zyz; Karkehabadi *et al.*, 2014 ▸) as the search model. For cross-validation, 5% of the X-ray diffraction data were excluded from the refinement for *R*_free_ calculations (Brünger, 1992 ▸). Throughout the refinement, the electron-density maps were inspected and the model was manually adjusted during repetitive cycles of iterative model building using *Coot* v.0.8.3 (Emsley *et al.*, 2010 ▸) and maximum-likelihood refinement using *REFMAC* v.5.8.0135 (Murshudov *et al.*, 2011 ▸). Water molecules were added using *ARP*/*wARP* v.7.1 (Lamzin & Wilson, 1993 ▸). Statistics from data processing and structure refinement are summarized in Table 1 ▸. Figures were produced using *PyMOL* (DeLano, 2002 ▸) and *Plot* (Wesemann, 2007 ▸). The secondary-structure elements were assigned using *STRIDE* (Heinig & Frishman, 2004 ▸). Sequence similarities were calculated with *ClustalW* (Larkin *et al.*, 2007 ▸). Root-mean-square deviation values (r.m.s.d.s) were calculated using *LSQMAN* (Kleywegt, 1996 ▸). Protein-interface volumes were calculated using *PISA* (Krissinel & Henrick, 2007 ▸). Atom coordinates and structure factors have been deposited in the Protein Data Bank (PDB) with accession code 5ju6.

## 3. Results and discussion   

### 3.1. ReCel3A production and purification   

The GH3 BGL *Re*Cel3A was heterologously produced in an *H. jecorina* strain with the four major cellulase genes (*cbh1*, *cbh2*, *egl1* and *egl2*) deleted. In this background *Re*Cel3A was the major protein as judged by SDS–PAGE analysis. This simplified the subsequent purification steps in comparison to the previous production of *Re*Cel3A in a wild-type *H. jecorina* strain (Murray *et al.*, 2004 ▸). *Re*Cel3A was purified to homogeneity using a custom affinity column. The *p*-aminobenzyl-β-d-glucose affinity matrix was an efficient step to separate *Re*Cel3A from background proteins.

### 3.2. Hydrolysis of lignocellulosic biomass   

**Figure 1:**
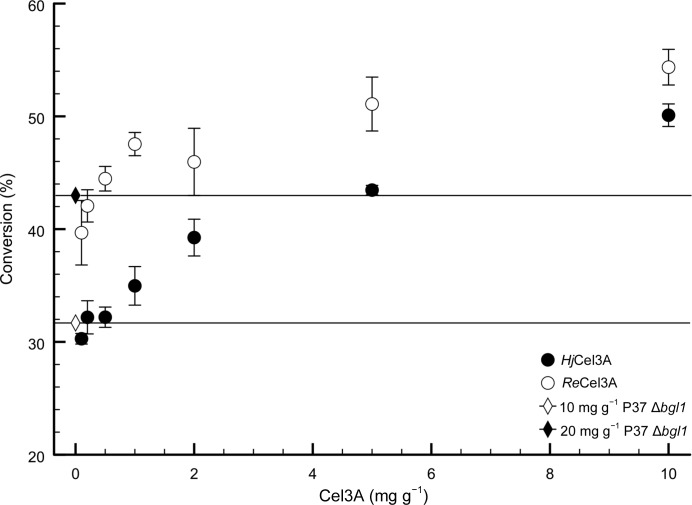
Saccharification of washed acid-pretreated corn stover with 10 mg g^−1^ *H. jecorina* strain P37 Δ*bgl1* supplemented with 0.1–10 mg g^−1^ β-glucosidases. Data points and error bars represent the mean and standard deviation of four replicates. Horizontal lines indicate the conversion levels of 10 and 20 mg g^−1^ P37 Δ*bgl1* as indicated.

Previously, we demonstrated that *H. jecorina* cellulase mixtures with increased levels of the native BGL *Hj*Cel3A have enhanced cellulose-degradation activity (Karkehabadi *et al.*, 2014 ▸). In this study, *H. jecorina* P37 Δ*bgl1* whole cellulase (lacking *Hj*Cel3A) mixtures supplemented with increasing levels of BGL (either *Re*Cel3A or *Hj*Cel3A) were compared for the degradation of PCS (Fig. 1 ▸). The mixtures containing *Re*Cel3A showed an up to 25% increase in glucose release compared with mixtures with an equal amount of *Hj*Cel3A added. These results encouraged us to study *Re*Cel3A in more detail biochemically and to solve its three-dimensional structure using X-ray crystallography.

### 3.3. Enzyme kinetics   

The accumulation of cellobiose during enzymatic biomass degradation severely inhibits the activity of cellulases, especially glycoside hydrolase family 7 cellobiohydrolases (Bezerra *et al.*, 2006 ▸; Gruno *et al.*, 2004 ▸). We have previously shown that increasing the amount of endogenous *Hj*Cel3A in mixtures of *H. jecorina* whole cellulase increases the conversion of phosphoric acid-swollen cellulose and washed PCS to glucose (Karkehabadi *et al.*, 2014 ▸). The interest in the enzyme *Re*Cel3A stemmed from the initial biochemical characterization performed by Murray *et al.* (2004 ▸). In this study it was shown that *Re*Cel3A was a relatively thermostable GH3 BGL and retained much of its activity even at higher temperatures. We investigated the enzymatic properties of *Re*Cel3A on different soluble glucan substrates, as reported in Table 2 ▸. The highest catalytic efficiency of *Re*Cel3A among the substrates tested was for hydrolysing 2-chloro-4-nitrophenyl-β-d-glucopyranoside. More interestingly, there was a higher *k*_cat_/*K*_m_ towards cellobiose over cellotriose. Hrmova *et al.* (1998 ▸) have previously shown that the barley BGL ExoI (*Hv*ExoI) has an increased affinity towards longer cellodextrins. This is also the case for *Hj*Cel3A (Karkehabadi *et al.*, 2014 ▸), which when combined with their reported broad substrate affinity indicates that hydrolysing accumulating cellobiose during the degradation of cellulose might not be the primary or the only biological function of GH3 BGLs. The catalytic efficiencies of *Re*Cel3A for the hydrolysis of model substrates, as found by Murray *et al.* (2004 ▸), are in general lower than those found for *Hj*Cel3A (Karkehabadi *et al.*, 2014 ▸). We also compared the melting temperatures of *Re*Cel3A and *Hj*Cel3A (Table 3 ▸). *Re*Cel3A has an 8°C higher melting temperature compared with *Hj*Cel3A in the presence of 1 m*M* glucose. The superior performance of *Re*Cel3A on PCS could be explained by its apparent preference for cellobiose compared with other types of disaccharides and cellodextrins and potentially by its higher thermal stability compared with *Hj*Cel3A.

### 3.4. Crystallization, structure solution and model building   

**Figure 2:**
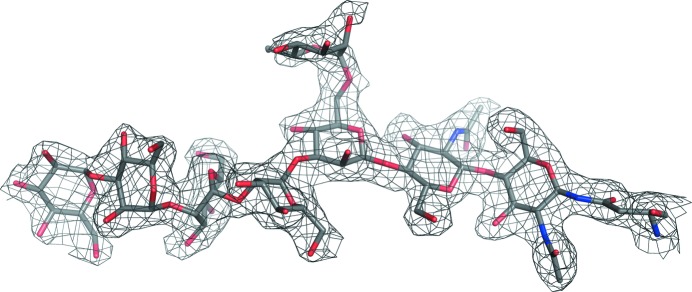
Electron density for glycosylation II, an incorrectly trimmed glycosylation chain, Glc_1_Man_5_GlcNAc_2_, at Asn249.

Purified and concentrated *Re*Cel3A crystallized in the orthorhombic space group *P*2_1_2_1_2_1_, with refined unit-cell parameters *a* = 137.3, *b* = 148.6, *c* = 196.4 Å. The molecular-replacement solution obtained using *Phaser* (McCoy *et al.*, 2007 ▸) gave the best solution with four protein molecules (MW = 90.4 kDa) in the asymmetric unit, with a calculated *V*_M_ of 2.77 Da^−1^ (Matthews, 1968 ▸) and an estimated solvent content of 56%. Initial phases were obtained using *H. jecorina* Cel3A (*Hj*Cel3A; PDB entry 3zz1; Karkehabadi *et al.*, 2014 ▸) as a search model. The electron-density map obtained after molecular replacement was of very good quality, from which it became obvious that the protein was heavily glycosylated (Fig. 2 ▸). The *Re*Cel3A structure at 2.2 Å resolution was refined to final *R*_work_ and *R*_free_ values of 18.8 and 23.8%, respectively. The final *Re*Cel3A structure model, consisting of four non­crystallographic symmetry (NCS)-related *Re*Cel3A molecules in the asymmetric unit, contains a total of 3348 amino-acid residues, 1842 water molecules and a total of 181 carbohydrate residues. The structure model contains 32 *cis*-peptides, and there are 36 cysteines, of which 32 form 16 disulfide bonds. Additional X-ray data-collection and refinement statistics for the *Re*Cel3A structure model are presented in Table 1 ▸.

### 3.5. Overall structure   

The *Re*Cel3A crystal structure model is composed of four NCS-related *Re*Cel3A protein molecules. The average root-mean-square deviation between the four molecules in the *Re*Cel3A structure is 0.2 Å (with a highest deviation of 0.22 Å and a lowest deviation of 0.17 Å). Each of these protein chains consists of 834 amino-acid residues, and the first and last visible residues in all four *Re*Cel3A molecules in the crystal structure are Asp21 and Pro855, respectively, of the translated deposited *Re*Cel3A DNA sequence (GenBank AAL69548.3). Residues 1–20 of the translated *Re*Cel3A DNA sequence constitute the signal peptide, as predicted by the *SignalP* server (Petersen *et al.*, 2011 ▸), and are cleaved off prior to secretion of the mature protein. The numbering of amino-acid residues in the *Re*Cel3A structure model starts from Met1 of the pre-protein. Each one of the four NCS-related *Re*Cel3A molecules consists of three distinct structure domains, which are connected by two linker regions. No electron density is visible for the C-terminal residues 856–857, probably owing to high flexibility in this region of the protein. All other amino-acid residues of the four NCS-related *Re*Cel3A molecules in the structure model are well ordered, and no gaps in the electron density for the main-chain atoms are found.

**Figure 3:**
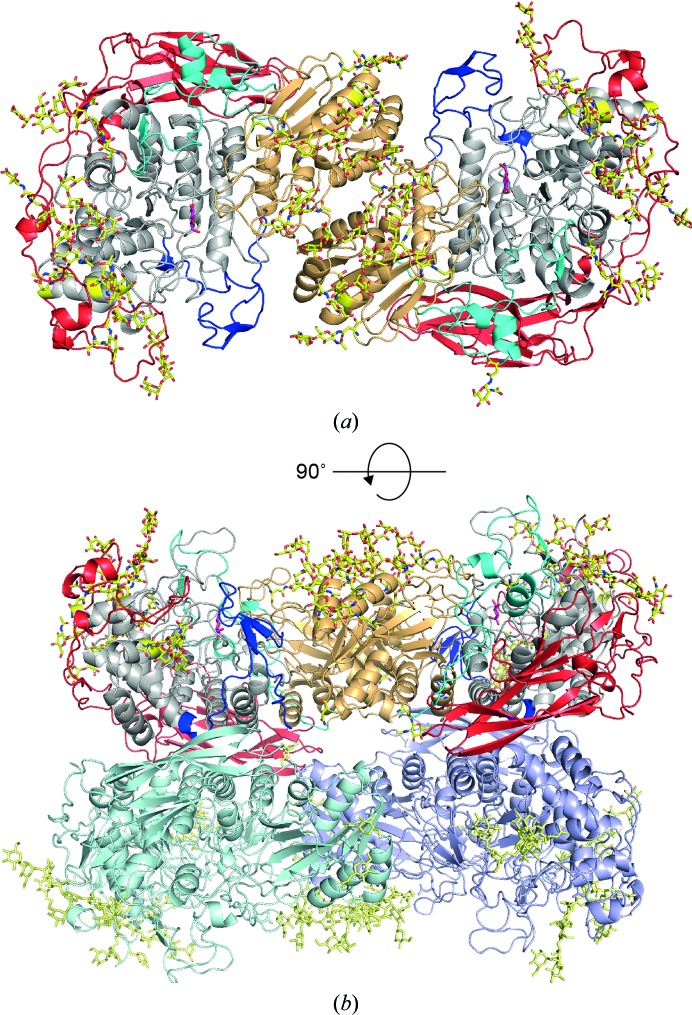
(*a*) Cartoon representation of the *R. emersonii* Cel3A (*Re*Cel3A) dimer. The N-terminal domain is coloured light grey, the first linker dark blue, the second domain gold, the second linker cyan, the C-terminal domain red and the N-linked glycosylations yellow; the glucose in the −1 subsite is depicted in magenta. (*b*) The view is rotated 90° compared with (*a*) and now shows the tetrameric assembly of *Re*Cel3A in the asymmetric unit. The two protein chains in the second dimer are coloured light teal and light purple. N-linked glycosylations are depicted in light yellow.

The *Re*Cel3A structure is a three-domain structure with an assembly that is similar to previously reported structures, with an N-terminal domain with a TIM-barrel-like ββ(β/α)_6_ fold, sometimes also referred to as a collapsed TIM barrel, a middle (α/β)_6_ sandwich domain (coloured gold in Fig. 3 ▸), which contains the glutamic acid that acts as a general catalytic acid, and a third C-terminal domain (coloured red in Fig. 3 ▸) with an fibronectin type III-like (FnIII-like) fold.

### 3.6. Overall fold   

There is a large diversity in the domain composition of the available GH3 structures. Most of the currently available three-dimensional structures of GH3 proteins have the canonical TIM-barrel domain in common. The exceptions are fungal β-glucosidases such as *Re*Cel3A, *Hj*Cel3A (Karkehabadi *et al.*, 2014 ▸), *A. aculeatus* BGLI (Suzuki *et al.*, 2013 ▸), *Af*βG and *Ao*βG (Agirre *et al.*, 2016 ▸) and also *K. marxianus* BglI (Yoshida *et al.*, 2010 ▸) and the bacterial β-glucosidase *Tn*Bgl3B (Pozzo *et al.*, 2010 ▸), which all share the ββ(β/α)_6_ fold. These enzymes also have a third FnIII-like domain in common, the presence of which might contribute to stabilizing the fold of the first ββ(β/α)_6_-fold domain and allow the otherwise stable TIM barrel to collapse during evolution and open up for changes around the catalytic centre.

### 3.7. Subsite −1 and catalytic residues   

**Figure 4:**
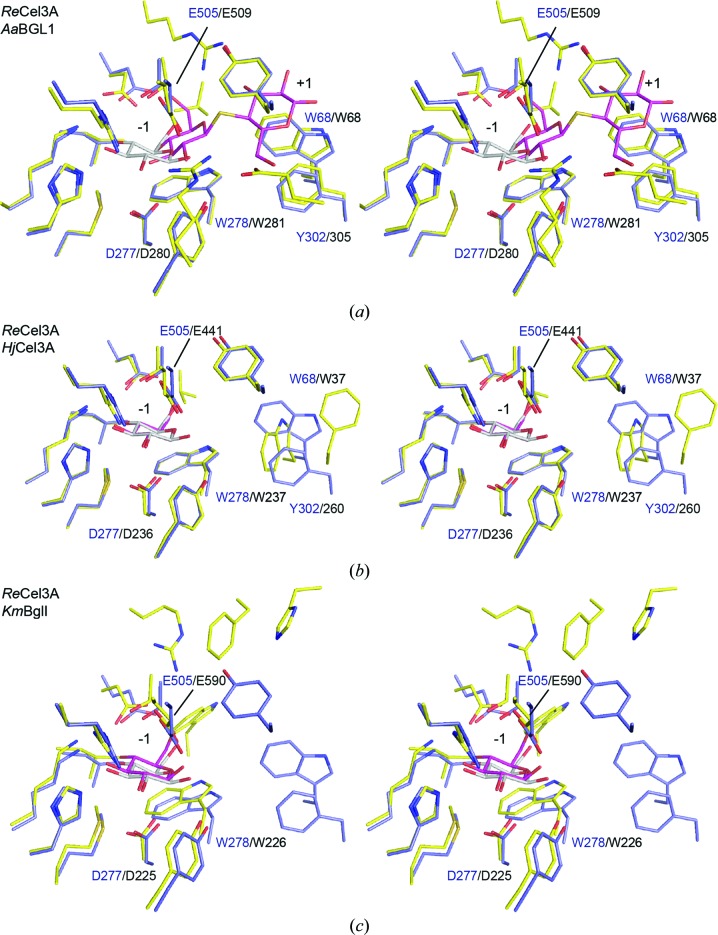

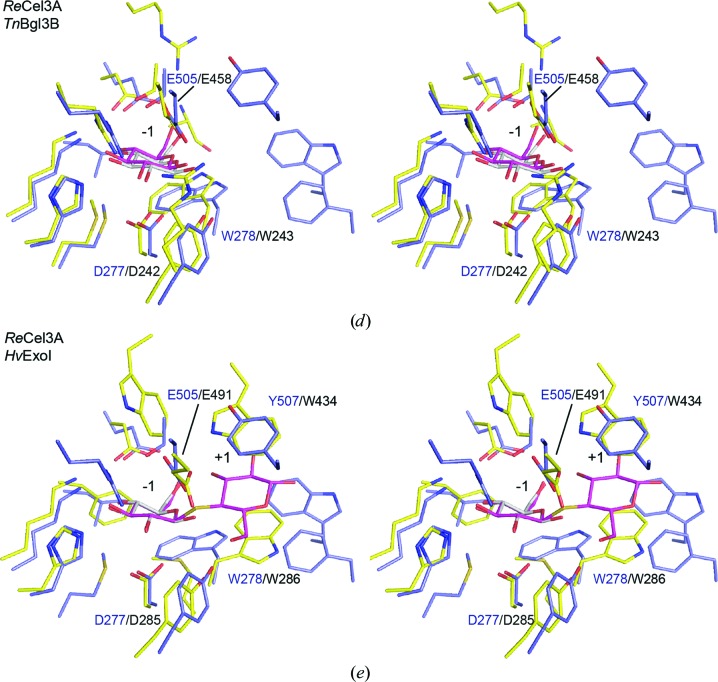
Stereo stick representations of the catalytic centres of GH3 β-glucosidases. *R. emersonii* Cel3A is shown in yellow and the −1 glucose monosaccharide in grey; ligands from aligned structures are shown in magenta. (*a*) *A. aculeatus* BGL1 (*Aa*BGL1; PDB entry 4iig; Suzuki *et al.*, 2013 ▸) in blue, (*b*) *H. jecorina* Cel3A (*Hj*Cel3A; PDB entry 3zyz; Karkehabadi *et al.*, 2014 ▸), (*c*) *K. marxianus* BglI (*Km*BglI; PDB entry 3ac0; Yoshida *et al.*, 2010 ▸), (*d*) *T. neapolitana* Bgl3B (*Tn*Bgl3B; PDB entry 2x41; Pozzo *et al.*, 2010 ▸) and (*e*) *H. vulgare* ExoI (*Hv*ExoI; PDB entry 1iex; Hrmova *et al.*, 2001 ▸).

The large number of hydrogen bonds between the protein and the ligand bound in the catalytic centre makes the binding in subsite −1 of GH3 enzymes highly specific. The two catalytic residues in *Re*Cel3A were identified based on homology to other GH3 structures: Asp277 (nucleophile) and Glu505 (acid/base) (Fig. 4 ▸). In the *Re*Cel3A structure, clear density is observed for a glucose unit in the −1 subsite and no indication of distortion from the relaxed chair conformation can be observed.

### 3.8. Putative +1 subsite   

Trp278 of the *Re*Cel3A structure aligns in the sequence with Trp268 of *Hv*ExoI, which is one side of the proposed ‘coin slot’ (Varghese *et al.*, 1999 ▸). Trp278 has a similar inward shift towards the −1 subsite as the corresponding tryptophan residues in *Aa*BGL1, *Hj*Cel3A, *Km*BglI and *Tn*Bgl3B (Figs. 4 ▸ *a*–4 ▸ *d*). The inward shifting of the tryptophan residue breaks the ‘coin slot’ and the rearrangement is a direct consequence of the collapsed TIM barrel described above. The −1 subsite widens when the second barrel β-strand is shorter and antiparallel. One side of the proposed ‘coin slot’ in the +1 subsite is partially replaced by the Tyr507 side chain. Alhough originating from another loop in the second domain, the phenolic ring occupies almost the same space as the benzene ring of the ‘coin slot’ tryptophan (Trp434 of HvExo1) to narrow the +1 subsite and the entrance to the active site (Fig. 4 ▸ *e*).

Next to Trp278 in *Re*Cel3A and potentially replacing the other side of the ‘coin slot’ are the two conserved aromatic residues Phe302 and Trp68. When compared with the corresponding residues in *Hj*Cel3A, the plane of the Trp68 side chain has turned almost 90° away from the +1 subsite. This allows aromatic stacking of the Phe302 and Trp68 side chains to form a hydrophobic ‘knob’ and places the phenylalanine residue in the +1 subsite rather than in the +2 subsite as in *Hj*Cel3A. This aromatic side-chain stacking further narrows the +1 subsite and contributes to a less pronounced +2 subsite. Although these aromatic residues are present in many BGLs this stacking is not observed in *Hj*Cel3A, while it is in all three *Aspergillus* β-glucosidases with known structure [*Aa*BGL1 (Figs. 4 ▸ *a* and 4 ▸ *b*), *Af*βG and *Ao*βG].

Previously, we have shown that *Hj*Cel3A prefers the hydrolysis of slightly longer oligosaccharides, *i.e.* of cellotriose and cello­tetraose compared with cellobiose (Karkehabadi *et al.*, 2014 ▸). Our data for *Re*Cel3A show that this enzyme prefers cellobiose to cellotriose. There is no increase in activity on cellotriose compared with cellobiose, which indicates that the +2 subsite contributes relatively little to substrate recognition. For *Hj*Cel3A, the activity increased for cellotriose compared with cellobiose, thus indicating the importance of a +2 subsite for *Hj*Cel3A (Karkehabadi *et al.*, 2014 ▸). As mentioned above, the presence of a +2 subsite is less pronounced in *Re*Cel3A than in *Hj*Cel3A, where the phenylalanine is also complemented with an asparagine (Asn261 in *Hj*Cel3A) to form the +2 subsite. The lack of a +2 subsite in *Re*Cel3A could explain the activity profile for the enzyme as a more pronounced cellobiase than *Hj*Cel3A.

### 3.9. Dimerization   

**Figure 5:**
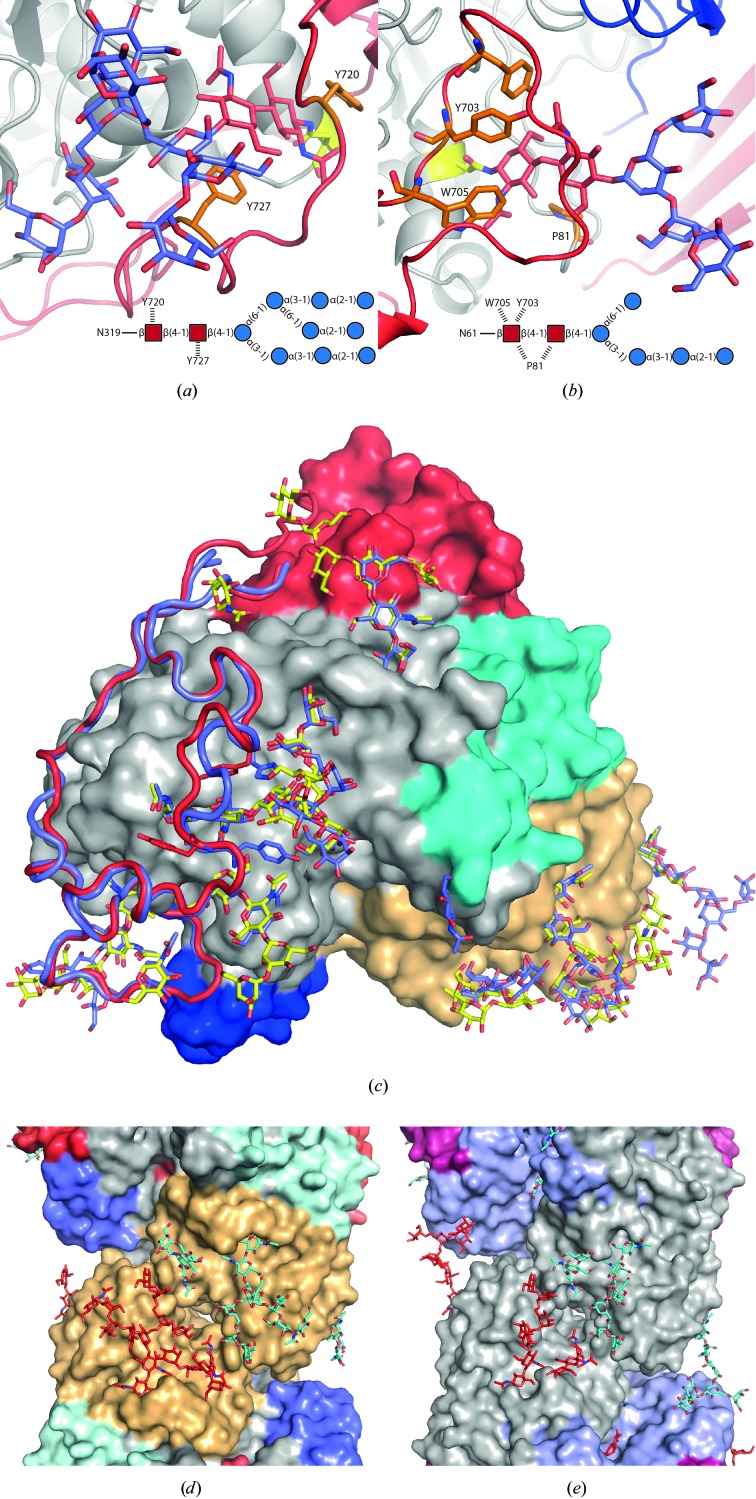
(*a*, *b*) Cartoon representations of the *R. emersonii* Cel3A (*Re*Cel3A) N-glycosylation chains IV and I (blue and light red) that are covered by the extended C-terminal loop (red) in the structure. (*c*) Surface representation of *Re*Cel3A with the corresponding two N-glycosylations coloured yellow. The extended C-­terminal loops of *Re*Cel3A (red) and *A. aculeatus* Bgl1 (*Aa*Bgl1; PDB entry 4iig) are shown as ribbons (blue). *Aa*Bgl1 N-­glycosylations, in stick representation, are coloured blue. In (*d*) and (*e*) surface representations of the dimer interfaces of the *Re*Cel3A and *Aa*Bgl1 structures are shown, respectively. N-­glycans from the two lower molecules are coloured red and those from the two upper molecules in teal.

It has been shown that *Re*Cel3A forms dimers in solution (Murray *et al.*, 2004 ▸), which was confirmed in this study when performing gel-filtration characterization of *Re*Cel3A. Dimerization is clearly supported by the crystal structure and also by the structure of *Aa*BGL1 (Suzuki *et al.*, 2013 ▸). The two molecules in the dimer are related by a 180° rotation (Fig. 3 ▸ *a*). The dimer interface has a total overall contact area of 1572 Å^2^, to which the modelled N-glycans contribute about 19%. It is mainly formed between the (α/β)_6_ sandwich domains, but also includes interactions between the sandwich domain and the linker between domains 1 and 2 (Fig. 5 ▸ *d*). This is similar to what is found in the *Aa*BglI structure (Fig. 5 ▸ *e*), which has a contact area of 1450 Å^2^, but including the modelled N-glycans the contact area increases to 1935 Å^2^. Similar interaction surfaces were observed in the recent publication by Agirre *et al.* (2016 ▸) on the structures of two *Aspergillus* β-glucosidases, one of which crystallized as a tetramer although with a slightly different architecture than that observed for *Re*Cel3A.

### 3.10. N-glycosylation   

*Re*Cel3A is highly N-glycosylated, with a pattern resembling those of the three β-glucosidases from *Aspergillus* (Agirre *et al.*, 2016 ▸; Suzuki *et al.*, 2013 ▸). There are a total of 16 glycosylation sites in *Re*Cel3A with the Asn-*X*-Ser/Thr N-glycosylation sequon. The *Re*Cel3A structure model contains a total of 181 glycosylation residues, as summarized in Table 4 ▸. In spite of this relatively generous glycosylation of the *Re*Cel3A molecules, it was possible to crystallize the protein without enzymatic removal of the N-glycans prior to the crystallization experiments. A large number of carbohydrate chains attached to the *Re*Cel3A molecules in the structure model can be observed and modelled, and the longest chain is composed of ten carbohydrate residues. We can also see that the glycosylation chains contribute interactions at the crystal contacts as well as between the two NCS-related molecules.

In *Re*Cel3A we can model carbohydrate chains, such as Man_7_GlcNAc_2_, that are known to be prevalent in Rut-C30-derived strains of *H. jecorina* (Stals *et al.*, 2004 ▸). Wild-type strains of *H. jecorina* show a more normal endoplasmic reticulum (ER) glycosylation trimming, yielding Man_5–6_GlcNAc_2_ chains. Such glycans are the result of the trimming of Glc_3_Man_9_GlcNAc_2_, which is then transferred to the nascent peptide chain in the ER by α-glucosidases found in the ER. Further trimming occurs normally by the action of α-mannosidases and β-*N*-acetylglucosaminidases. The Rut-C30-derived strains have an in­efficient ER-α-glucosidase, which accounts for the presence of untrimmed monoglycosylated N-glycans (Stals *et al.*, 2004 ▸). We can clearly observe both the longer incompletely trimmed glycan chains, Man_8_GlcNAc_2_, and shorter monoglycosyl­ated Glc_1_Man_5_GlcNAc_2_ and Man_5–6_GlcNAc_2_ chains (Fig. 5 ▸ *a*), as well as single *N*-acetyl­glucosamine (GlcNAc) residues, in the *Re*Cel3A structure. Modelled carbo­hydrate glycans are not in themselves evidence of an N-glycosylation pattern. It is expected that glycosylation chains that are flexible and are not restrained by the protein crystal packing will not be observable using crystallo­graphic methods. However, single GlcNAc residues are observed in the *Re*Cel3A tetramer that cannot be part of a longer glycan as they pack tightly between protein chains and presumably provide important crystal contacts.

**Figure 6:**
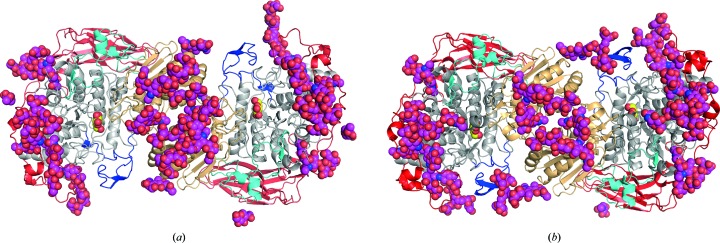
Cartoon representations of (*a*) the *R. emersonii* Cel3A (*Re*Cel3A) and (*b*) the *A. aculeatus* BGLI (*Aa*Bgl1) dimers in the two structures. N-glycans in the two structures are shown as magenta spheres.

The exact mechanisms of how glycosylations affects the structure and function of cellulases and other proteins are unknown. N-glycosylation has been shown to increase the solubility, reduce the aggregation and enhance the thermal stability of proteins (Wang *et al.*, 2010 ▸; Kayser *et al.*, 2011 ▸; Ioannou *et al.*, 1998 ▸). In *Re*Cel3A most of the larger glycans reside on the first domain (chains I–IV). Chains V and VII are situated on the second domain, close to the proposed dimer interface of *Re*Cel3A. The N-glycosylation chain IV shows the remarkable feature of being buried by the extended C-terminal loop. Two conserved aromatic residues, Tyr720 and Tyr727, on the loop provide stacking interactions with the two buried NAG residues 1201 and 1202. This is very similar to what was reported for the two *Aspergillus* β-glucosidases (Agirre *et al.*, 2016 ▸). As stated previously, *Re*Cel3A is most likely to exist as a dimer in nature. Interestingly, the overall glycosylation pattern for the dimer shows that the active site on each of the monomers seems to be encircled by glycans, of which some originate from the other monomer (Fig. 6 ▸ *a*). Interestingly, the opposite face of *Re*Cel3A is seemingly devoid of glycosylation (Fig. 3 ▸ *a*), both modelled glycans and predicted sites, with the notable exception of glycan chain VI in protein chains *A* and *C*, where a single GlcNAc interacts with the opposite NCS-related protein molecule. The glycosylations in *Re*Cel3A could be a contributing factor to the thermal stability of the enzyme. Another function could be to protect the active site from lignin-derived aromatic compounds or to promote substrate binding. It has been shown that N-glycans bind aromatic residues (Yamaguchi *et al.*, 1999 ▸) and potentially cellulose (Payne *et al.*, 2013 ▸).

## 4. Conclusions   

The GH family 3 BGL Cel3A from *R. emersonii* is an efficient supplement to whole cellulase mixtures for the production of fermentable sugars from lignocellulosic biomass. *Re*Cel3A seems to have a preference for disaccharides over longer β-1,4-glucans, indicating a primary role as a cellobiose in the degradation of cellulosic biomass. The three-domain architecture of the *Re*Cel3A structure, the collapsed TIM-barrel, α/β sandwich and FnIII domain, also contains an extended C-terminal loop and a relatively high number of attached N-glycans. The majority of the attached glycans are either covered by the extended loops present in the *Re*Cel3A structure or are situated at the dimer interface between two *Re*Cel3A molecules. This might suggest that the glycans are functional in the sense of stabilizing the loop covering the collapsed TIM-barrel domain and possibly providing binding interactions at the dimeric interface. *Re*Cel3A exhibits a higher thermostability compared with *Hj*Cel3A, and enhances PCS saccharification compared with *Hj*Cel3A. These superior biochemical characteristics make *Re*Cel3A a potential candidate for replacing an enzyme such as *Hj*Cel3A in commercial enzyme mixtures for the conversion of lignocellulosic biomass into fermentable sugars. Owing to its thermal stability, *Re*Cel3A could be part of enzyme mixtures that operate at elevated process temperatures, potentially resulting in overall increased reaction rates for the process.

## Supplementary Material

PDB reference: Cel3A from *Rasamsonia emersonii*, 4d0j

## Figures and Tables

**Table 1:** Data collection and processing Values in parentheses are for the outer shell.

Data collection	
Space group	*P*2_1_2_1_2_1_
Unit-cell parameters (Å, °)	*a* = 137.3, *b* = 148.6, *c* = 196.4, α = β = γ = 90
X-ray source	ID23-1, ESRF
Wavelength (Å)	0.97625
Resolution (Å)	2.2
Resolution range (Å)	48–2.2
Total No. of observations	919229
Unique reflections	233437
〈*I*/σ(*I*)〉	11.4 (3.6)
*R*_merge_ †	0.11 (0.48)
Multiplicity	4.5 (4.5)
Structure refinement	
*R*_work_/*R*_free_ (%)	17.3/22.8
R.m.s.d., bond distances (Å)	0.015
R.m.s.d., bond angles (°)	1.18
No. of amino-acid residues	3340
No. of water molecules	1688
No. of sugar residues	187
Ramachandran plot‡	
Most favoured regions (%)	96
Allowed regions (%)	4
Disallowed regions (%)	0
Pyranose conformations (total/percentage)§	
Lowest energy conformation	187/100
Higher energy conformations	0.0/0

† *R*_merge_ = 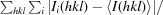 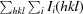, where *I_i_*(*hkl*) is the intensity of the *i*th measurement of an equivalent reflection with indices *hkl* and 〈*I*(*hkl*)〉 is the mean intensity of *I_i_*(*hkl*) for all *i* measurements.

‡ Calculated using a strict-boundary Ramachandran definition given by Kleywegt & Jones (1996 ▸).

§ Calculated using the *Privateer* software (Agirre *et al.*, 2015 ▸) within *CCP*4*i*2 and presented as introduced by Agirre *et al.* (2016 ▸).

**Table 2:** Kinetic parameters of *Re*Cel3A with chromophoric substrates and disaccharide oligosaccharides

Substrate	*K*_m_ (m*M*)	*k*_cat_ (s^−1^)	*k*_cat_/*K*_m_ (*M* ^−1^ s^−1^)
CNPG	0.40	14	3.6 × 10^4^
pNPG	0.40	5.4	1.4 × 10^4^
CNPX	0.66	0.23	3.5 × 10^2^
Cellobiose	0.78	5.5	7.1 × 10^3^
Cellotriose	0.39	0.72	1.9 × 10^3^

**Table 3:** Melting temperatures of *Hj*Cel3A and *Re*Cel3A in the presence and absence of glucose

β-Glucosidase	*T*_m_ (°C)
*Hj*Cel3A	77.6
*Hj*Cel3A + 1 m*M* glucose	79.0
*Re*Cel3A	87.3
*Re*Cel3A + 1 m*M* glucose	87.3

**Table 4:** N-glycosylation chains and IDs in *Re*Cel3A

Residue	Chain *A*	Chain *B*	Chain *C*	Chain *D*	ID
61	5	5	9	6	I
249	7	5	8	8	II
312	4	4	5	4	III
319	9	10	9	9	IV
438	4	5	4	3	V
470	1	—	1	1	VI
519	9	10	8	8	VII
532	1	1	1	1	VIII
538	1	—	2	1	IX
560	2	2	3	1	X
636	—	1	1	—	XI
707	1	—	—	1	XII
731	1	1	—	1	XIII
Total	45	44	50	44	
Overall total	183				
